# Wear-coping mechanisms and functional morphology of the radular teeth of *Vittina turrita* (Neritimorpha, Gastropoda)

**DOI:** 10.1098/rsif.2025.0016

**Published:** 2025-05-28

**Authors:** Wencke Krings, Ellen Schulz-Kornas, Stanislav N. Gorb

**Affiliations:** ^1^ Department of Cariology, Endodontology and Periodontology, University of Leipzig, Leipzig 04103, Germany; ^2^ Department of Electron Microscopy, University of Hamburg, Hamburg 20146, Germany; ^3^ Department of Functional Morphology and Biomechanics, Kiel University, Kiel 24118, Germany; ^4^ Department of Mammalogy and Paleoanthropology, Leibniz Institute for the Analysis of Biodiversity Change, Hamburg, 20146, Germany

**Keywords:** feeding structure, mechanical properties, abrasion, structural failure, interface, mollusc

## Abstract

In most molluscan species, the food is manipulated and taken in by the radula, a chitinous structure exhibiting diverse morphologies and compositions. The teeth of Patellogastropoda and Polyplacophora are well studied, with heavy mineralization reducing wear and failure. However, some gastropod taxa possess unmineralized teeth, even though they forage from rocks. This study characterizes the teeth of the gastropod *Vittina turrita* as representative neritid species. Using a combination of techniques—scanning electron microscopy, confocal laser scanning microscopy, nanoindentation and energy-dispersive X-ray spectroscopy —the biomechanical and compositional properties of the teeth were examined. The heterogeneous presence of compositional gradients, together with previous wear analyses, renders the teeth to have different functions. Some teeth are involved in loosening food, collecting food particles or, as joints, spanning the radula in a certain configuration. A key finding was the presence of tooth coatings enriched with calcium (Ca) in regions prone to abrasion. The study also identified heterogeneities in autofluorescence patterns, which were directly associated with the distribution of Ca within the coatings and the degree of tanning. This study broadens our understanding of mechanical adaptation in gastropod feeding structures, showing that feeding from solid surfaces is also possible with partial and targeted reinforcement instead of full tooth mineralization—and that structure–function relationships are more diverse than previously thought.

## 1. Introduction

The radula, the intricate and highly varied food-processing organ, enables molluscs to exploit a broad spectrum of food sources with diverse mechanical properties across the phylum, which can be considered a driving force behind species diversification and evolution. By studying the properties of different types of radulae from species foraging on different food sources, adaptations to the preferred food sources can be studied.

### 1.1. Radular morphology

**Figure 1.**
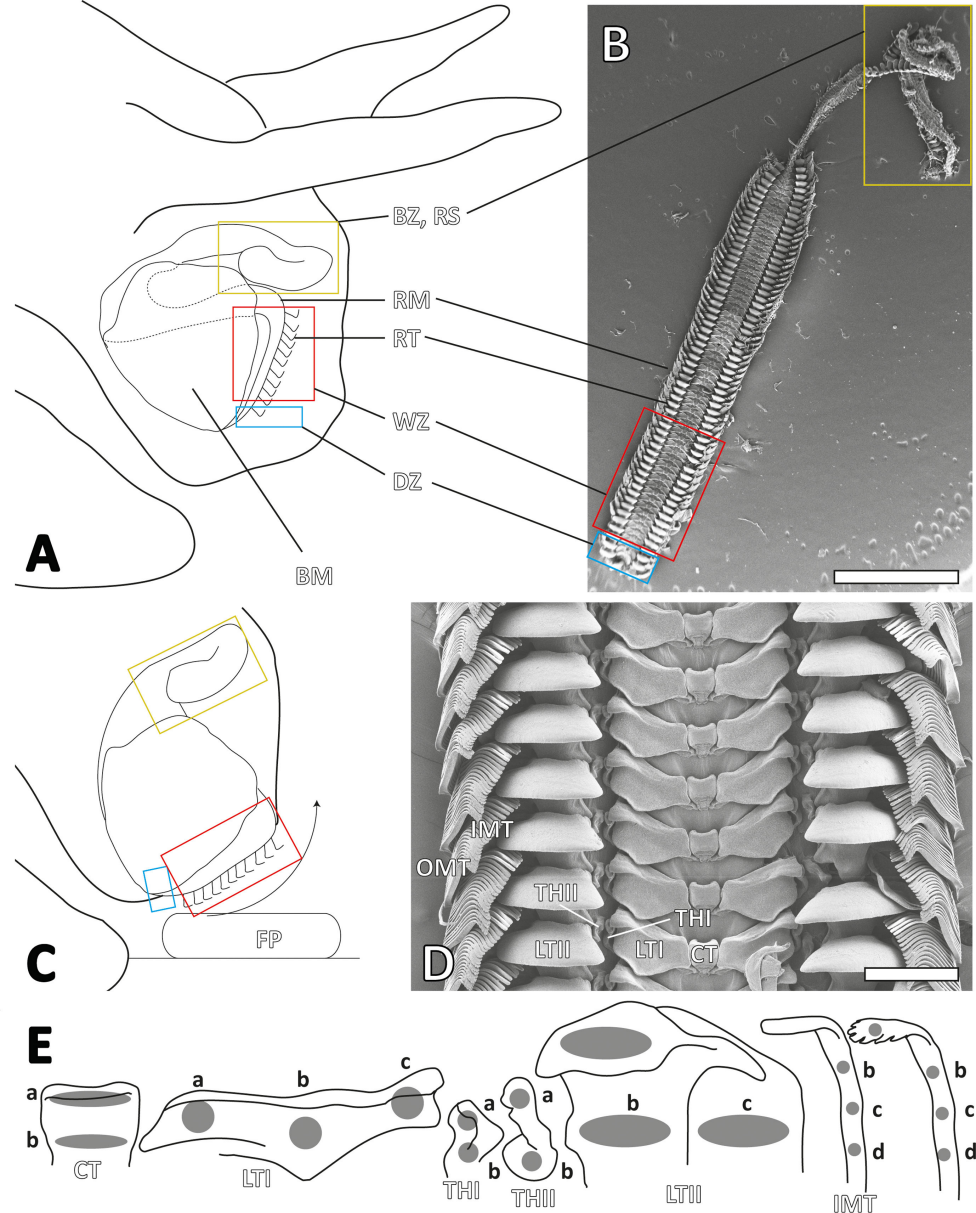
A. Schematic illustration of the buccal mass and radula in a gastropod. B. Scanning electron microscopy (SEM) image showing the entire radula of *Vittina turrita* with all ontogenetic zones (degenerative zone, working zone, building zone). C. Schematic illustration of the buccal mass and radula during feeding action. D. SEM image of the radular working zone with the different tooth types (central tooth, lateral tooth I, thickening of the membrane I and II, lateral tooth II, inner and outer marginal teeth). Orientation of the radula is different from that in B, as in malacological literature, the working zone is oriented upwards and the building zone downwards. The underlying buccal mass structures were removed by forceps. E. Tooth regions of interest for X-ray spectroscopy (EDX) and nanoindentation analyses. Abbreviations: BM, buccal mass; BZ, building zone (the area where new radular structures are secreted); CT, central tooth; DZ, degenerative zone; FP, food particle; IMT, inner marginal tooth; LTI, lateral tooth I; LTII, lateral tooth II; OMT, outer marginal tooth; RM, radular membrane; RS, radular sack; RT, radular tooth; THI, thickening of the membrane I or reduced medial tooth; THII, thickening of the membrane II or reduced lateral tooth; WZ, working zone (the active radular area where teeth engage with food particles). Scale bars: B, 2 mm; D, 200 µm.

The radula comprises a non-extensible chitinous membrane (e.g. [1]) with embedded rows of teeth. These teeth, in conjunction with underlying odontophoral cartilages, associated muscles, the alary processus and in some taxa the jaw, form the feeding apparatus known as the buccal mass (figure 1A).

The arrangement and number of similarly shaped teeth per transverse row became key for the categorization of radulae into approximately a dozen basic types. This approach provided a framework for managing molluscan diversity as these types often follow phylogeny (e.g. [2–5]). One prominent radular type includes the rhipidoglossan radulae, found in Vetigastropoda and Neritimorpha, characterized by numerous small marginal teeth, one larger central tooth and several lateral teeth (figure 1D,E).

### 1.2. Radular composition

Interactions between ingesta at the radular working zone (figure 1A–D), the active area used for foraging, induce tooth wear, as radular teeth must transfer high forces onto hard target surfaces enduring significant stress concentrations (e.g. [6–15]). The radular supporting structures, such as odontophoral cartilages and radular bolsters, contribute to stress reduction by acting as cushions or muscular hydrostats (e.g. [13,14,16–19]).

However, although the radula is continuously regenerated by posterior epithelia in the radular sac or building zone (figure 1A–C) [7,20–24], some structural adaptations also minimize wear and prevent structural failure during interaction with ingesta.

For instance, species that perform scratching actions often show mechanical property gradients along the teeth, with hard or stiff cusps and soft or flexible bases, helping the mitigation of stress concentrations (e.g. [25]). This has been tested in Polyplacophora [26–29], limpets [30], Cephalopoda [15], paludomid gastropods [31–37] and nudibranch gastropods [38]. These gradients increase strain at the tooth base and reduce stress at the tips, enabling the teeth to act effectively on ingesta while bending under excessive stress. This bending also allows adjacent teeth to interlock in some taxa [25,39,40], distributing stress across multiple teeth—a phenomenon termed the ‘collective effect’, which has been experimentally studied recently [29,41,42]. These mechanical property gradients result from several factors. One of them is the distribution of inorganic compounds (e.g. minerals or metal ions concentrated in the cusp), which is known from Polyplacophora [11,26,29,43–48] and Patellogastropoda [49,50]. Additionally, the protein distribution, the degree of protein tanning and cross-linking influence the properties. This has been studied in limpets [1,30,51], Polyplacophora [29,52], paludomid gastropods [32,35–37,53] or nudibranch gastropods [38] (for cross-linking mechanisms, see [54]). Also, the chitin fibre density and architecture [55–59] and the water content in the teeth and radular membrane [29,41,42] determine the property gradients.

In taxa, such as Polyplacophora [26,29,43–48], Cephalopoda [15] and Patellogastropoda (e.g. [11,49,50,58]), Paludomidae [35,59], Cephalaspidea [38] and some Nudibranchia [60,61], wear-coping mechanisms also include the incorporation of high levels of iron (Fe), calcium (Ca) or silicon (Si) in the superficial regions of teeth (coating). This incorporation produces harder cusps capable of withstanding abrasive ingesta, such as Porifera spicules, crustacean carapaces, Foraminifera or algae attached to stone surfaces. In Polyplacophora and Patellogastropoda, dominant teeth have cusps filled with these elements, whereas in cephalopod, paludomid, heterobranch and nudibranch taxa, a thin outer layer with high Ca or Si concentrations covers the cusps, reducing abrasion.

However, despite existing research, wear- and failure-coping mechanisms remain understudied, specifically in species with non-mineralized teeth. To gain more insight into this topic, we here focus on the neritid gastropod *Vittina turrita* (Gmelin, 1791), which possesses a rhipidoglossan radula. *Vittina turrita* feeds on algae from solid substrates, but also ingests from softer substrates such as wood [62]. In a previous work, we documented the three-dimensional position of the radular teeth during feeding by experiments involving sandpapers of different roughness [14]. We found that some teeth were heavily affected by abrasion (the lateral tooth II, the inner marginal teeth, the lateral edge of the lateral tooth I). Yet, other teeth did not show wear after feeding from sandpaper (the central teeth, the outer marginal teeth, the medial side of the lateral tooth I, the thickenings of the membrane I and II). It was shown that a complex folding of the membrane during feeding leads to a close interaction of some teeth with the substrate, whereas other teeth do not interact with the substrate and probably act as joint-like structures that support the folding and unfolding mechanisms [14]. Here, we add the knowledge on the material properties of the teeth by testing the local mechanical properties by nanoindentation, which allows us to develop hypotheses on tooth biomechanical behaviour and function. The material heterogeneity was studied by confocal laser scanning microscopy (CLSM), which documents the material autofluorescence after excitation with four different lasers. The resulting autofluorescence is, however, difficult to interpret without determining the elemental composition, as heavy mineralization is known to affect the signal. To test for high mineral content, energy-dispersive X-ray spectroscopy (EDX) analyses were included. This also allowed us to test for correlations between mechanical properties and elemental composition. Previously [53,63], we tested the inner tooth structure by EDX, but it was not related to the mechanical properties. Additionally, here we add data on the outer tooth coating.

### 1.3. Hypotheses

We hypothesize (a) that the tooth regions, which previously demonstrated close interaction with the substrate, presumably should have higher mechanical property values to potentially reduce failure and wear. (b) These mechanical properties are potentially related to the content of inorganic compounds. We propose that (c) the regions of high interaction also possess a coating with a higher content of inorganic compounds as potential adaptation to reduce wear.

## 2. Material and methods

### 2.1. Specimens and preparation

Adult individuals of *V. turrita* were acquired from an online pet shop (available at garnelio.de, here gastropods are sold as *Neritina turrita*) and identified [62]. For consistency, only individuals of uniform size (shell height 18−20 mm) were included in the study, as the buccal mass size is known to vary with the body size (e.g. [64,65]). In this study, we used 16 radulae overall.

The entire buccal mass was carefully extracted using forceps. Supporting structures were carefully removed from the radular membrane with forceps. Each radula was placed in an Eppendorf tube containing 70% ethanol and cleaned of organic tissues by a short ultrasonic bath for 5 s.

### 2.2. Scanning electron microscopy

For imaging, four radulae were mounted on scanning electron microscopy (SEM) sample holders using double-sided adhesive carbon tape and coated with a 5 nm layer of platinum (Pt). SEM imaging was performed with a Zeiss LEO 1525 microscope (One Zeiss Drive, Thornwood, USA) at 5 kV. Two radulae were mounted on SEM sample holders and the teeth were broken by tweezers to gain insight into the material microstructure.

### 2.3. Confocal laser scanning microscopy

To document structural heterogeneities, we followed the protocol by Michels & Gorb [66], which utilizes the autofluorescence of arthropod cuticle when exposed to lasers of different wavelengths. Although primarily applied to arthropod cuticle, this method has also been successfully used for chitinous structures with higher mineral content, such as crustacean cuticle [67–69] and radular teeth [15,32,36–38,60,61].

For this study, two cleaned radulae were mounted on glass slides, each surrounded by a stack of reinforcement rings. These rings were filled with glycerine (greater than or equal to 99.5%, water-free; Carl Roth GmbH & Co. KG, Karlsruhe, Germany) and sealed with a glass coverslip. Autofluorescence from different regions of the radular working zone was visualized using a Zeiss LSM 700 confocal laser scanning microscope (Carl Zeiss Microscopy GmbH, Jena, Germany) equipped with four solid-state lasers at wavelengths of 405, 488, 555 and 639 nm. Bandpass or longpass emission filters (420–480, greater than or equal to 490, greater than or equal to 560 and greater than or equal to 640 nm, respectively) were applied. The resulting autofluorescence images were superimposed using maximum intensity projection in Zeiss Efficient Navigation (ZEN) software (Carl Zeiss MicroImaging GmbH, Jena, Germany). Colours were assigned to signals from different lasers as follows: blue for 405 nm, green for 488 nm, red (50% saturation) for 555 nm and red (50% saturation) for 639 nm.

In unmineralized chitinous structures, autofluorescence signals correlate with the following material properties: (i) sclerotized, stiff material produces a red signal (555 and 639 nm excitation), (ii) weakly sclerotized chitin generates a green signal (488 nm excitation), and (iii) high elastic protein content or unsclerotized chitin corresponds to a blue signal (405 nm excitation) (e.g. [32,60,66,70]). For structures with higher inorganic content, green may correspond to silica and blue to the calcium content [36–38,60,61,67–69]. To interpret the CLSM images, we investigated teeth by elemental analysis.

### 2.4. Elemental analysis

To analyse the elemental composition using EDX, 10 additional radulae were extracted and cleaned in an ultrasonic bath with distilled water for 1 min.

To perform EDX analyses on the tooth coating facing the oral cavity, we attached 10 radulae to SEM sample holders with double-sided adhesive carbon tape as flat as possible. Afterwards, samples were coated with a 5 nm layer of Pt. Elemental composition was assessed using a Zeiss LEO 1525 SEM equipped with an Octane Silicon Drift Detector (SDD) (microanalysis system TEAM, EDAX Inc., New Jersey, USA). Consistent imaging parameters were used for all samples, including an acceleration voltage of 20 kV. The detector was calibrated using a clean, plain copper plate affixed to a clean aluminium sample holder prior to analysis; this included elemental mapping performed at 20 kV and lasting 1 min.

The detected elements were Al (aluminium), C (carbon), Ca (calcium), Cl (chlorine), Fe (iron), H (hydrogen), Mg (magnesium), N (nitrogen), Na (sodium), O (oxygen), P (phosphorus), Pt (platinum), S (sulfur) and Si (silicon). Some elements, such as H, C, N and O (key components of chitin and proteins) and Al and O (from polishing powder), were measured, but not described further. Due to an overlap between the P and Pt peaks, P content could not be reliably determined, and these were analysed together as PPt. To establish a baseline for Pt content, 15 areas of pure epoxy resin were tested, yielding a mean ± standard deviation of 0.13 ± 0.04 atomic %. This confirmed that none of the discussed elements (e.g. Ca, Cl, S, Si) originated from the epoxy, ruling out artefacts from sample preparation.

For analyses of the inner structure of the teeth, the radulae were first detached from the adhesive carbon tape using 70% ethanol, and the Pt coating was removed via a 30 s ultrasonic bath in 70% ethanol. The radulae were mounted on glass slides with double-sided adhesive carbon tape, following established protocols [32]. Each radula was encircled with a small metallic ring, which was then filled with epoxy resin (Reckli Epoxy WST, RECKLI GmbH, Herne, Germany) to fully encapsulate the radula. After polymerization at room temperature for three days, the glass slides and adhesive tape were removed.

The samples were polished using sandpapers of varying roughness until the radular tooth cross sections were exposed. The surfaces were then further smoothened with a suspension of aluminium oxide polishing powder (0.3 μm grain size; PRESI GmbH, Hagen, Germany) applied using a polishing machine (Minitech 233/333, PRESI GmbH, Hagen, Germany) to minimize electron scattering. The samples were cleaned again in an ultrasonic bath for 5 min to remove residual polishing powder, mounted onto SEM sample holders and coated with a 5 nm layer of Pt.

In total, 3200 small regions (0.1−0.2 × 0.1−0.2 μm) were successfully tested on predefined tooth parts (see figure 1E) from the working zone (per tooth region, 160 successful tests were analysed).

### 2.5. Nanoindentation

Nanoindentation experiments were conducted on the same 10 embedded radulae, the same teeth from the working zone and the same tooth regions (see figure 1E) used for the EDX analyses, following established protocols [31,34]. These tests focused exclusively on the inner structure of the embedded and polished teeth, as the native teeth were too roundish to test them directly with nanoindentation. For this reason, we have no mechanical property data on the tooth coating.

A nanoindenter SA2 (MTS Nano Instruments, Oak Ridge, USA) equipped with a Berkovich indenter tip and a dynamic contact module head was used. A total of 3200 measurement regions were successfully tested (per tooth region, 160 successful tests were analysed). All experiments were conducted under controlled room conditions (relative humidity 28−30%, temperature 22−24°C). The allowable drift rate was 0.1 nm s^−1^. A Poisson ratio of 0.3 was used. This ratio depends on the direction of testing, material composition (i.e. mineral presence) and water content. Our material is not purely elastic nor purely plastic, but in between. In the literature, a Poisson ratio of 0.3 is generally accepted for chitinous structures (e.g. [71,72]), and specifically for chitinous radular teeth [30]. The effective Young’s modulus (*E*) was calculated via the Oliver–Pharr method [73]. Hardness (*H*), the measure of the material’s resistance to plastic deformation, and *E*, the measure of the material’s elastic response to loading, were derived from force–displacement curves using the continuous stiffness mode. Measurements were taken at penetration depths of 600−800 nm. At each indentation site, approximately 200 values were collected across varying depths and averaged to obtain one *H* and one *E* mean value per test.

Because not all regions of interest were exposed simultaneously, a sequential process was employed. First, the initially visible target areas were analysed by EDX, followed by nanoindentation. Afterwards, the samples were further polished to expose the next regions of interest. EDX analyses were repeated, followed by additional nanoindentation tests, and this process continued until all targeted regions were evaluated.

### 2.6. Statistical analyses

All statistical analyses were conducted using JMP Pro v. 14 (SAS Institute Inc., Cary, NC, 1989−2007). Mean values with standard deviations were calculated for the EDX and nanoindentation results.

The Shapiro–Wilk W-test was applied to assess the normality of the data distribution. Since the data did not follow a normal distribution, a Kruskal–Wallis/Wilcoxon test was performed, followed by pairwise comparisons using the Wilcoxon method. These tests were used to compare the hardness (*H*) and Young’s modulus (*E*) between the regions of the different teeth. Additionally, correlation coefficients, estimated by the row-wise method, between the measured elements and mechanical properties were calculated to evaluate relationships.

## 3. Results

### 3.1. Radular morphology

**Figure 2.**
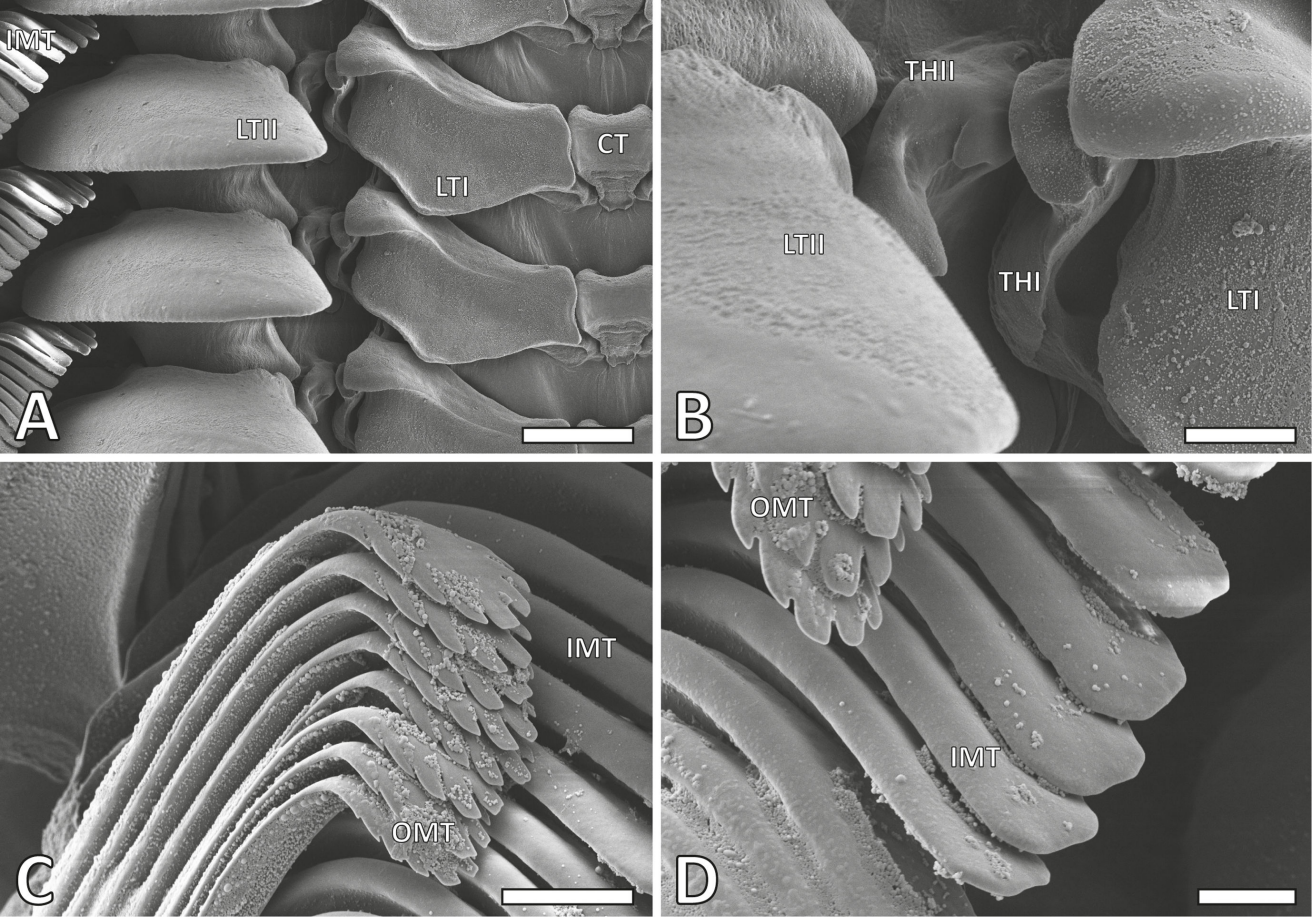
SEM images of the radula of *Vittina turrita*. A. Magnifications of the central and lateral teeth. B. Magnifications of the thickening of the membrane I and II. C. Magnifications of the outer marginal teeth. D. Magnifications of the inner marginal teeth. Abbreviations are the same as given in figure 1. Scale bars: A, 80 µm; B, C, 20 µm; D, 10 µm.

Each transverse row includes a central tooth (rachidian tooth), flanked on each side by one lateral tooth (lateral I), two either reduced teeth [74] or thickenings of the membrane (thickening of membranes I and II), a larger and more prominent lateral tooth (lateral II) and approximately 40 slender marginal teeth (15 inner and 25 outer marginal teeth) (figure 1D). The central tooth is almost rectangular, whereas the lateral tooth I features a broad, shoulder blade-shaped base with a thickened edge (figure 2A). The thickenings of membranes I and II, between the lateral tooth I and the lateral tooth II, interlock and are rather small and not easily identified (figure 2B). The lateral tooth II has a rounded shape with a broad base and a stylus merging into a thick cusp that bears one larger denticle and several smaller ones (figure 2A). Each marginal tooth consists of a stylus and a cusp. The inner 15 marginal teeth carry none or few small denticles (figure 2D), whereas the thinner and shorter outer 25 carry numerous and thinner denticles (figure 2C).

### 3.2. Microstructure

**Figure 3.**
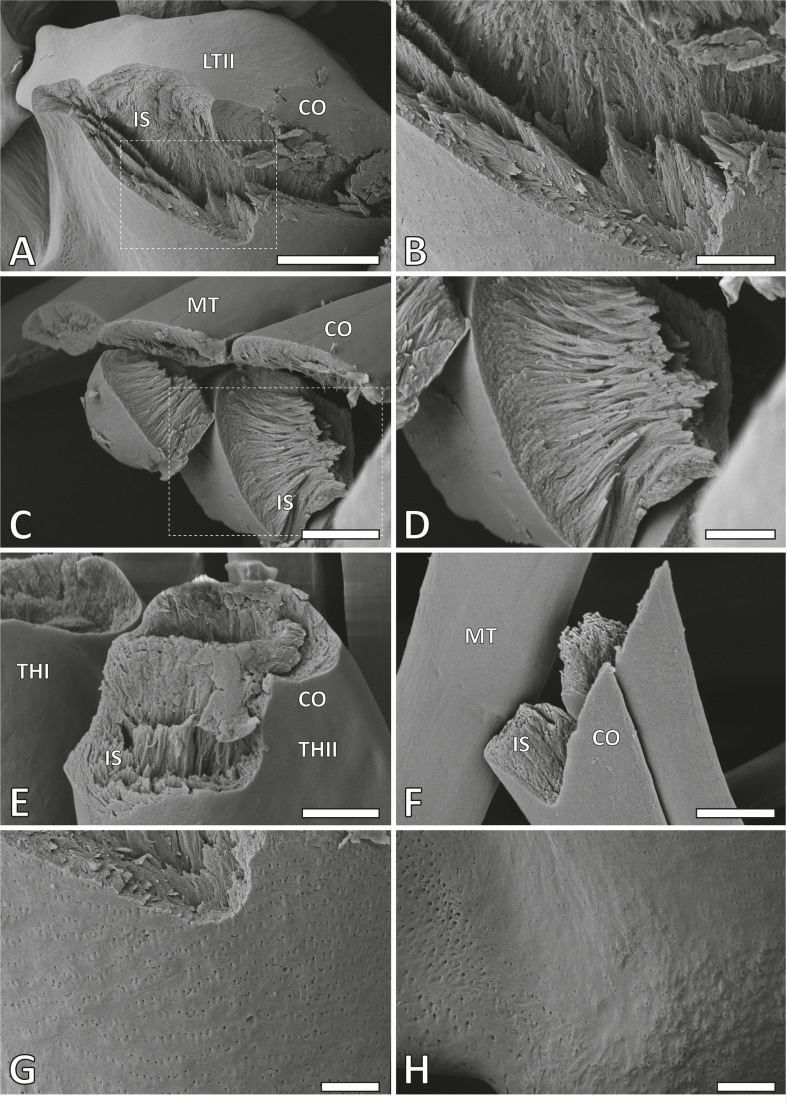
SEM images of broken radular teeth from the working zone displaying the fibrous inner tooth structure and the uniform, fibre-free outer coating. A,B. Lateral tooth II. C,D. Inner marginal teeth. E. Thickening of the membrane I and II. F. Outer marginal tooth. G. Surface of the lateral tooth II stylus, showing holes. H. Surface of the lateral tooth II basis, showing small humps. Abbreviations: CO, outer tooth coating; IS, inner tooth structure. Remaining abbreviations are the same as given in figure 1. Scale bars: A, 20 µm; B, 6 µm; C, E−H, 4 µm; D, 2 µm.

The tooth surface was covered by an extremely thin layer (coating) devoid of fibres of approximately 0.2−0.3 µm in thickness (figure 3). On the outside, we documented either holes of approximately 0.1−0.2 µm in diameter (figure 3G) or small humps of approximately 0.2−0.3 µm size with small holes (figure 3H). The holes were distributed across most tooth surfaces except the marginal teeth, whereas the humps were determined on the medial lateral tooth II basis.

The inner structures of the teeth were found to be nanoporous and composed of fibres (figure 3), each oriented from the tooth surface to the inside. The fibres appeared to be of similar size (approx. 0.01−0.03 µm) and changed orientation from the outer edge to the interior of the tooth. Near the surface, the fibres were mostly oriented perpendicular to the outer edges (figure 3C,D), while in the central tooth area, they seemed to be oriented along the tooth (from tooth cusp to basis).

### 3.3. Autofluorescence

**Figure 4.**
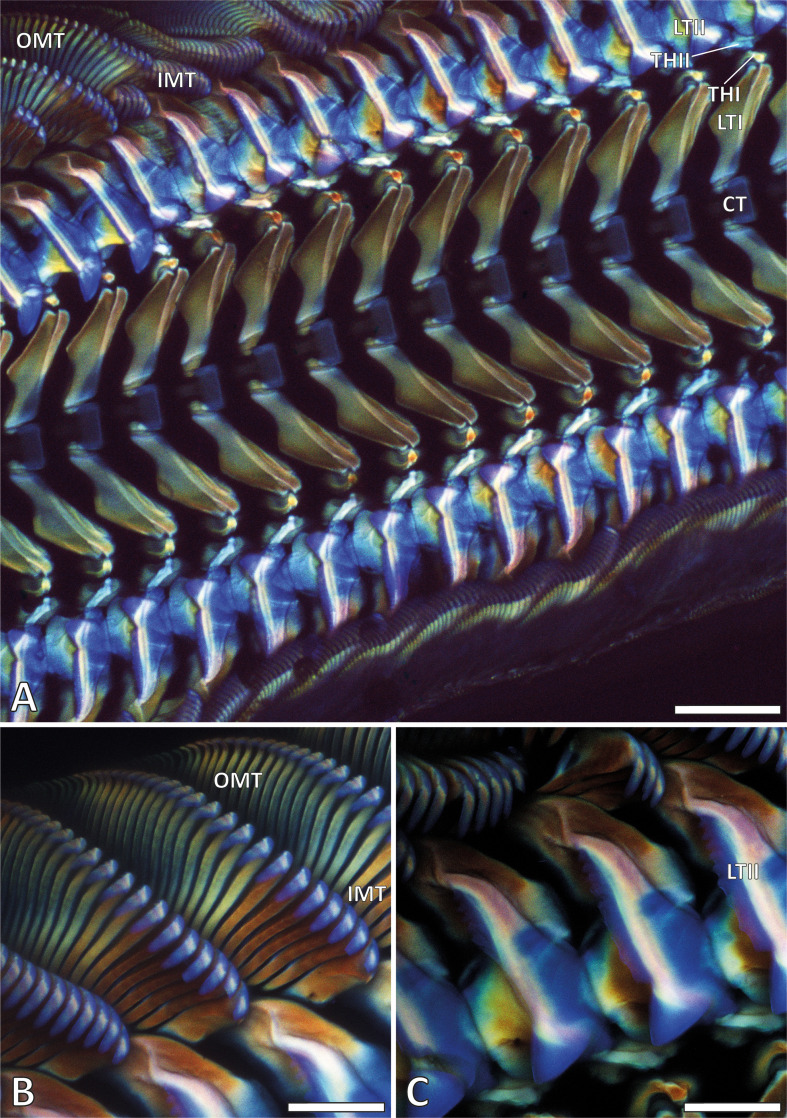
CLSM images of radulae. A. Working zone. B. Inner and outer marginal teeth of the working zone. C. Lateral teeth II. Abbreviations are the same as given in figure 1. Scale bars: A, 200 µm; B, C, 100 µm.

Mature teeth exhibited a consistent autofluorescence pattern (figure 4). The central teeth, the medial sides of the lateral teeth I, the cusps and the bases of the lateral teeth II, the cusps of the inner marginal teeth and the cusps and bases of the outer marginal teeth showed autofluorescence in response to the 405 nm wavelength laser, which was translated to blue in our images. These responses were, however, not as uniform as in the central teeth, the medial sides of the lateral teeth I and the bases of the outer marginal teeth, which showed a slightly different blue colour than the other regions.

The middle region of the lateral teeth I and the styli of the outer marginal teeth predominantly responded to the 488 nm wavelength laser, emitting a signal translated to green in our images (figure 4). The lateral sides of the lateral teeth I, the thickenings of the membrane I and II, the styli and lateral region of the cusps of the lateral teeth II and the styli of the inner marginal teeth exhibited autofluorescence signals in response to the 555 or 639 nm wavelength lasers, which were translated to red in our images.

### 3.4. Elemental composition

**Figure 5.**
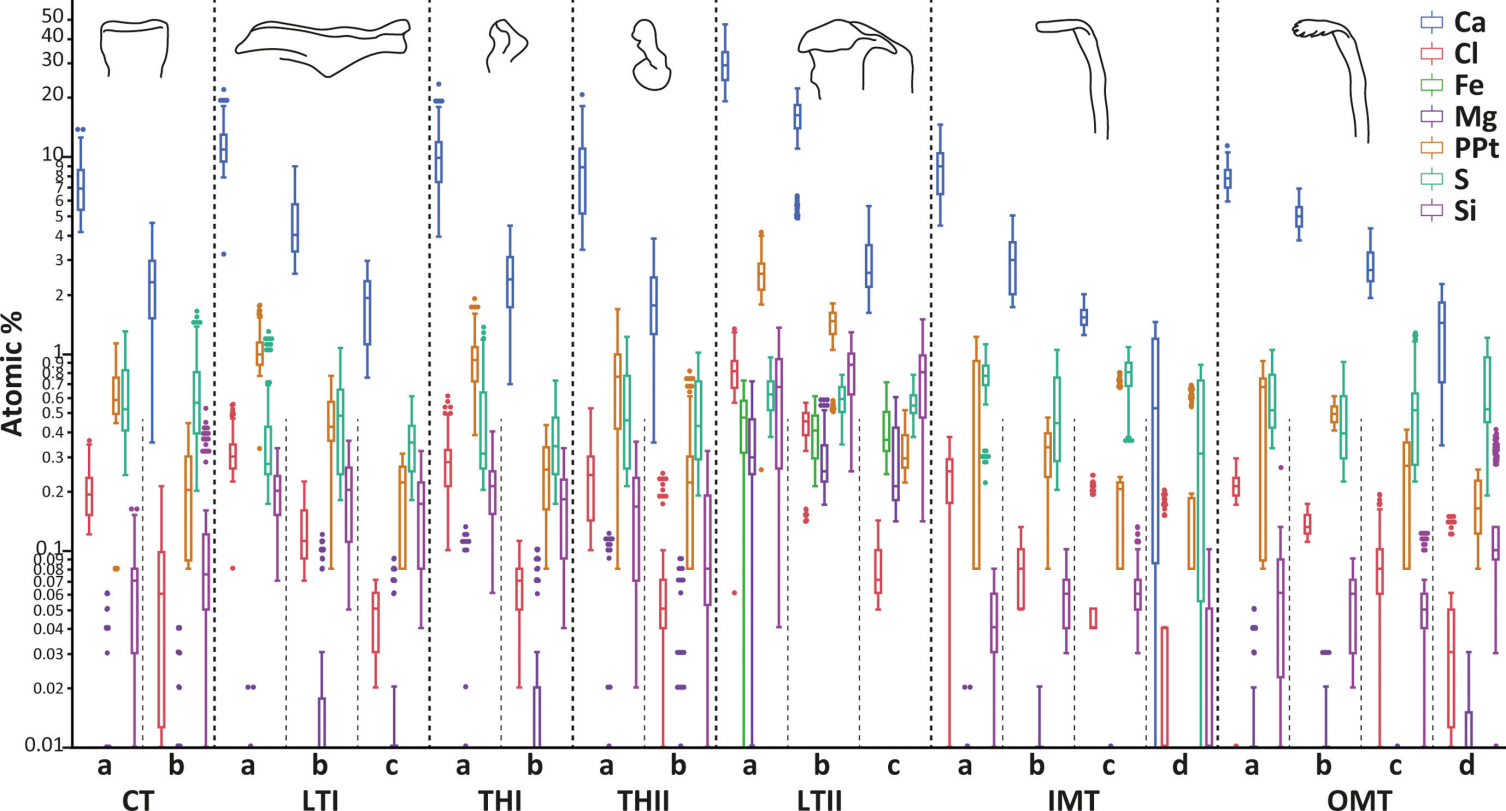
Results of the EDX experiments of the outer tooth coating. The elemental compositions (in atomic %) for the different regions of the distinct teeth are presented at a logarithmic scale. Abbreviations are the same as given in figure 1.

In the surface coatings of the structures (see figure 5, electronic supplementary material, figure S1 and table S1), Ca was regionally present in the highest proportions (mean values between 0.69 and 29.88 atomic %), followed by PPt (0.17−2.57). S was found with means of 0.36−0.77, Cl with 0.04−0.82 and Si with 0.03−0.81. Mg was only found in higher proportions in the lateral tooth II (means of 0.29−0.36). Fe was found exclusively in the lateral teeth II (means of 0.40−0.46). In general, the tooth regions, which were oriented towards the oral cavity (regions a), showed higher elemental content than the tooth regions, which were close to the membrane (regions c or d). Na was detected in very low proportions (0.00−0.04), without a clear distribution pattern.

**Figure 6.**
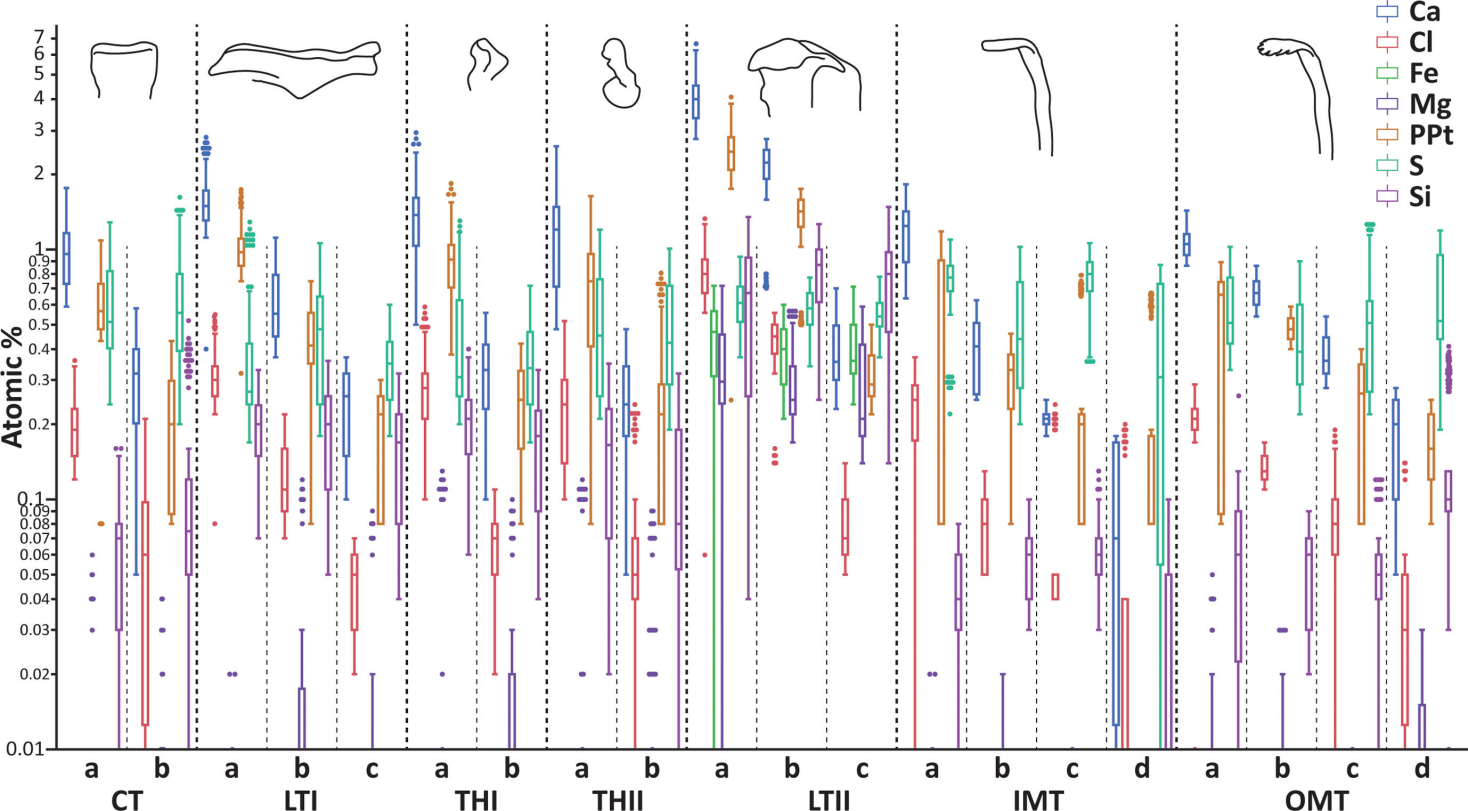
Results of the EDX experiments of the inner tooth structure. The elemental compositions (in atomic %) for the different regions of the distinct teeth are presented at a logarithmic scale. Abbreviations are the same as given in figure 1.

In the inner structures of the teeth (see figure 6, electronic supplementary material, figures S1 and table S2), Ca was regionally found in the highest proportions (mean values ranging from 0.25 to 4.05 atomic %), followed by PPt (0.19−2.50). S was present with means of 0.36−0.76, Cl with 0.06−0.81, and Si with 0.03−0.80. Mg was only found in higher proportions in the lateral tooth II (means of 0.28−0.36). Fe was found exclusively in the lateral teeth II (means of 0.39−0.46). In general, the tooth regions, which were oriented towards the oral cavity (regions a), showed larger elemental content than the tooth regions, which were close to the membrane (regions c or d). Na was detected in very low proportions (0.00−0.01), without a clear distribution pattern.

The elemental composition of the coatings and the inner structure were similar, with the exception of Ca, which was present in higher proportions in the outer tooth coating.

### 3.5. Mechanical properties

**Figure 7.**
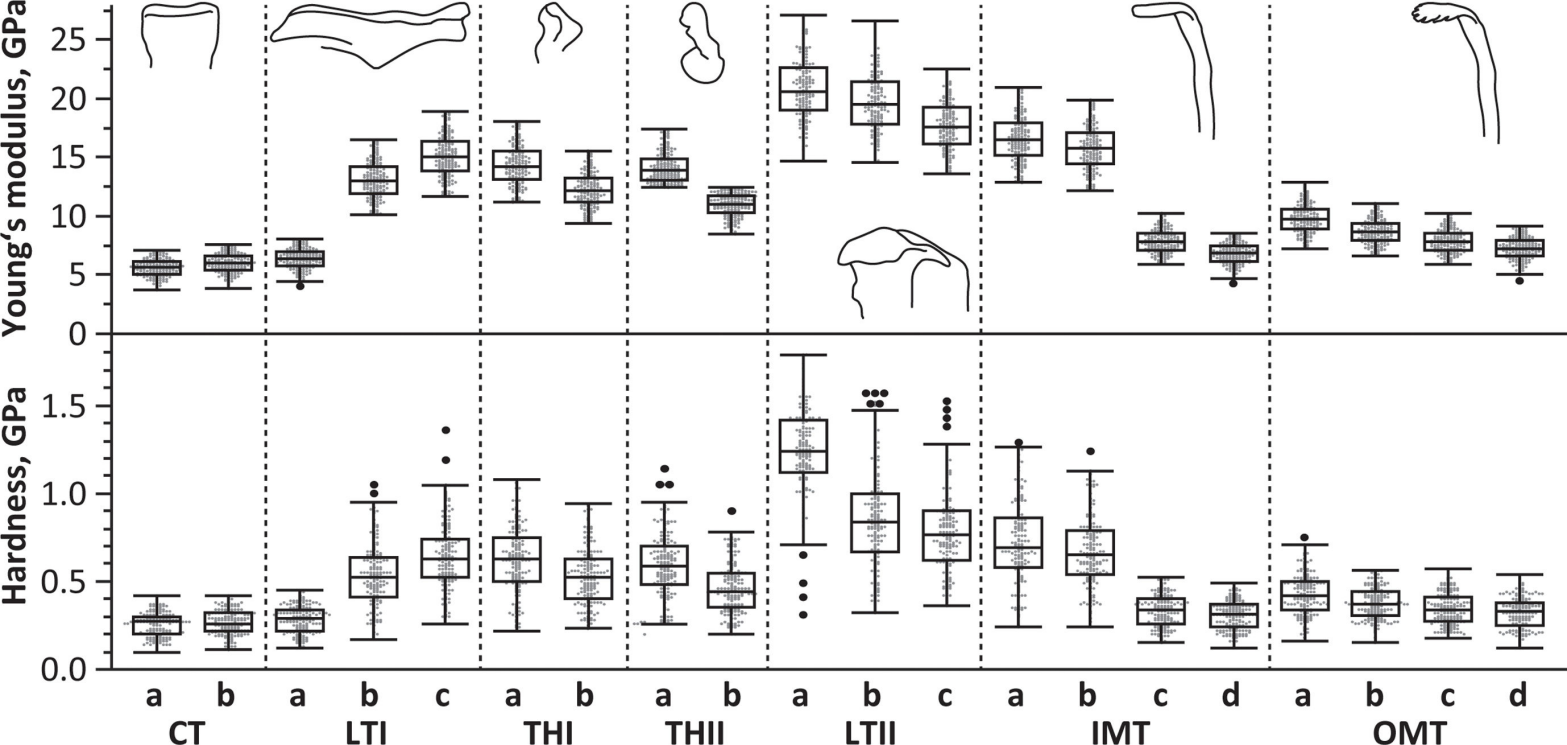
Results of the nanoindentation experiments. The effective Young’s modulus (*E*, in GPa) and hardness (*H*, in GPa) for the different regions of the distinct teeth. Abbreviations are the same as given in figure 1.

The parameters *E* (Young’s modulus) and *H* (hardness) exhibited a very strong positive correlation (*r* = 0.89). Mean values of *E* ranged from 20.75 to 5.61 GPa and mean values of *H* varied between 0.26 and 1.24 GPa (see figure 7 and electronic supplementary material, table S3). The lateral teeth II were the hardest and stiffest, followed by the inner marginal teeth, the thickening of the membrane I and II, laterals I, part of the lateral teeth I, the outer marginal teeth and finally the central teeth, which were the softest and most flexible ones. Within most tooth types, we determined mechanical property gradients (see figure 7 and electronic supplementary material, table S3). The only exception was the central tooth, where the region towards the oral cavity (region a) was rather similar to the region towards the membrane (region b). In the lateral teeth I, the lateral part (region c) was the hardest and stiffest one, followed by the central part (region b), followed by the medial part (region a) as the softest and most flexible region. In both thickenings of the membrane, the regions towards the oral cavity (regions a) were harder and stiffer, whereas the regions towards the membrane (regions b) were softer and more flexible. In the lateral teeth II, the tooth cusps were the hardest and stiffest regions, followed by the medial part of the tooth stylus (region b), and finally by the lateral part of the tooth stylus (region c). In the inner and outer marginal teeth, the tooth cusps (region a) were the hardest and stiffest regions, followed by the styli (regions b and c), and finally by the tooth bases (regions d).

Most regions differed significantly with regard to *H* and *E* values (*p* < 0.0001; for detailed *p*-values, see electronic supplementary material, tables S4 and S5). The regions that were not different were mostly regions of similar function (regions c, d in the outer marginal and the inner marginal tooth; region a in the thickening of the membrane I and II). In one other case, this revealed that the specific tooth was rather homogeneous in mechanical properties (central tooth).

### 3.6. Correlation coefficients and relationships

**Figure 8.**
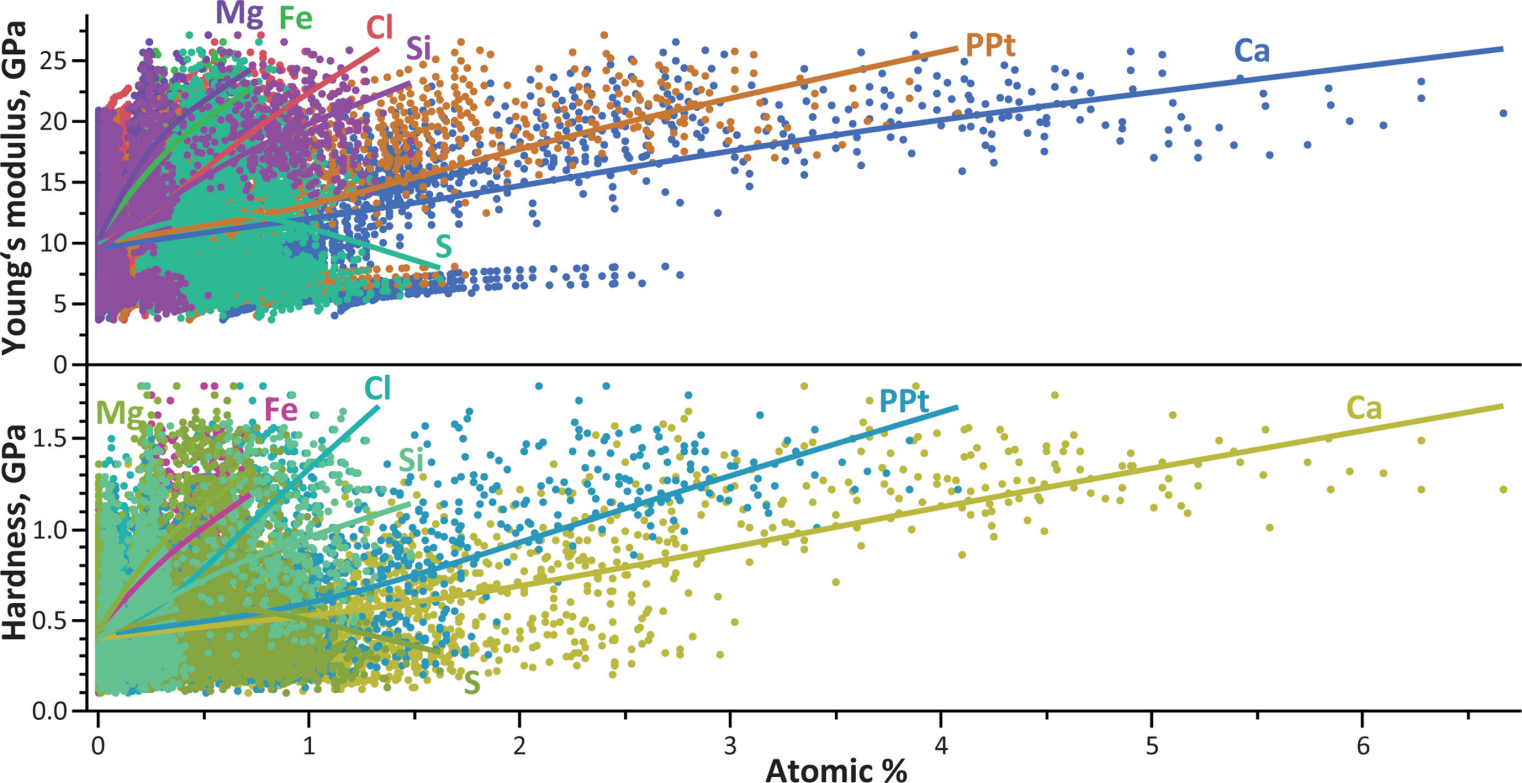
Relationship between the elements (given in atomic %) and the hardness (*H*) and effective Young’s modulus (*E*) (both given in GPa). The amount of Ca shows a clear positive relationship with the mechanical properties.

Ca and Cl (*r* = 0.99), PPt and Cl (*r* = 0.96), PPt and Ca (*r* = 0.95), Mg and Fe (*r* = 0.87), Si and Mg (*r* = 0.83) and Si and Fe (*r* = 0.74) exhibited strong positive correlations (see electronic supplementary material, table S6 for correlation coefficients and figure 8 for relationships). Moderate positive correlations were observed between PPt and Mg (*r* = 0.60), PPt and Fe (*r* = 0.59), Mg and Ca (*r* = 0.58), Mg and Cl (*r* = 0.58), Fe and Ca (*r* = 0.57) and Fe and Cl (*r* = 0.57). Low positive correlations were found for PPt and Si (*r* = 0.50), Si and Ca (*r* = 0.47) and Si and Cl (*r* = 0.46). Additionally, slightly positive correlations were noted for Na and Fe (*r* = 0.33) and Mg and Na (*r* = 0.21). Negligible positive correlations include those between Si and Na (*r* = 0.20), PPt and Na (*r* = 0.18), Ca and Na (*r* = 0.14), Na and Cl (*r* = 0.14), Fe and S (*r* = 0.10), Cl and S (*r* = 0.09), S and Na (*r* = 0.09), S and Ca (*r* = 0.08), S and Mg (*r* = 0.05), S and Si (*r* = 0.04) and S and PPt (*r* = 0.03).

With regard to the relationship between the mechanical properties and the elemental content, we could only test the inner structure as it was technically impossible to access data on the mechanical properties of the coating. *E* showed a moderate positive correlation to Fe (*r* = 0.62), Mg (*r* = 0.61), Si (*r* = 0.57) and Ca (*r* = 0.51) (see electronic supplementary material, table S6 for correlation coefficients and figure 8 for relationships). A moderate positive correlation was also found for *H* with Fe (*r* = 0.61), Mg (*r* = 0.60), Ca (*r* = 0.58), Cl (*r* = 0.57), PPt (*r* = 0.55) and Si (*r* = 0.51). Slightly positive correlations were noted for *E* and Cl (*r* = 0.50), PPt (*r* = 0.47) and Na (*r* = 0.21). Negligible positive correlations included *H* and Na (*r* = 0.16), *H* and S (*r* = 0.03) and *E* and S (*r* = 0.02).

Regions with high Ca concentrations in their coating (the lateral teeth II and cusps of the marginal teeth) exhibited strong autofluorescence in response to the 405 nm laser (translated to blue). In tooth regions without such a high concentration of Ca in the coating (the lateral sides of the lateral teeth I, the thickenings of the membrane I and II, the styli and lateral region of the cusps of the lateral teeth II and the styli of the inner marginal teeth), we detected that higher mechanical property values were measured in regions that exhibit a strong autofluorescence signal to the 555 or 639 nm wavelength lasers (translated to red). The central teeth, the medial sides of the lateral teeth I, the bases of the lateral teeth II and the bases of the outer marginal teeth were rather soft and flexible and showed autofluorescence in response to the 405 nm wavelength laser (translated to blue). The middle region of the lateral teeth I and the styli of the outer marginal teeth showed intermediate mechanical property values and also responded to the 488 nm wavelength laser (translated to green).

## 4. Discussion

### 4.1. Radular microstructure

The radula is a fibrous composite material characterized by a structural organization that mitigates fracture formation and propagation. It is composed of chitin fibres interwoven with proteins [1,75], forming an organic matrix with varied orientations, densities and arrangements. These diverse micro- and nano-architectures are probably adapted to functional loads: the external tooth material forms a relatively uniform coating, while underlying fibres are oriented perpendicular to the surface [11,49,59,75], enhancing resistance to both wear and fracture.

We found that in *V. turrita* teeth, the radular microstructure, with fibres running perpendicularly to the surface, resembles the fibre arrangement observed in limpets, chitons and paludomid gastropods [11,49,59,75–77]. We conclude that such fibre orientation probably enhances wear and fracture resistance, as the performance of biological materials composed of anisotropic structural units is heavily influenced by the orientation of these units. Anisotropy of units and its influence on wear and fracture resistance, outside the radular tooth realm, has been studied in plant branches (e.g. [78,79]) or pangolin scales (e.g. [80,81]). Typically, these units are aligned more parallel in the interior of the structure and become increasingly tilted towards the exterior, resulting in greater surface stiffness and improved fracture resistance at depth [82].

In addition, we found that the tooth surface was covered by an extremely thin coating devoid of fibres of approximately 0.2−0.3 µm in thickness. This situation is similar to other radular teeth [15,35–38,59–61], which possess a superficial hard and stiff layer to reduce wear. However, the thickness of the coating of *V. turrita* could vary between the tooth regions, but we were not able to measure the thickness of the entire coating with our methods.

On the outside of teeth, we documented either holes or small humps with holes. Possibly, the humps were present first and then eroded by interaction with the substrate. We assume that the holes are openings of channels or tubules, which deliver minerals to the tooth surface during tooth maturation; this, however, awaits further investigations.

### 4.2. Radular composition

**Figure 9.**
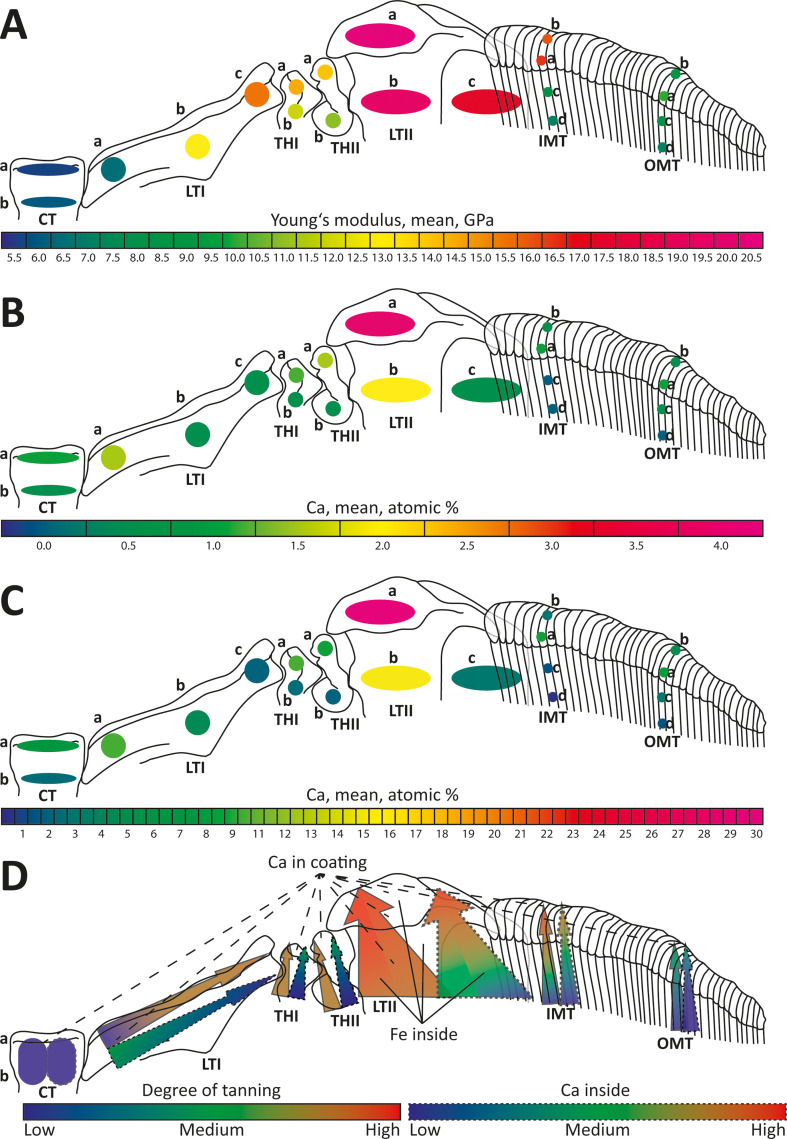
Teeth in three-dimensional position on the membrane during feeding, with the lateral tooth II as the highest structure and the central tooth as the lowest one. A. Mean Young’s modulus, given in GPa, plotted on the tested regions. B. Mean Ca, given in atomic %, of the inner structure plotted on the tested regions. C. Mean Ca, given in atomic %, of the tooth coating plotted on the tested regions. D. Interpretation on the origins of the mechanical property gradients. The arrows indicate the direction to which the mechanical properties increase, which is either towards the oral cavity or, for the lateral tooth I, towards the lateral direction. In most cases, the mechanical properties of the inner tooth structure seem to relate to the degree of tanning (black outline), but also to the content of Ca (dashed outline). In the lateral tooth II, Fe is exclusively found, which indicates that this element might be important for the cross-linking of proteins and increasing mechanical properties in this tooth type. In some tooth regions (indicated by black dashed lines), which are either oriented towards the oral cavity or, for the lateral tooth I, towards medial direction, we found a high content of Ca. Abbreviations are the same as given in figure 1.

In general, we found that the mechanical property gradients in the teeth of *V. turrita* are based on multiple mechanisms (figure 9D): the degree of tanning, as well as the Ca, P, Si, Fe and Mg content. In addition, we detected a tooth coating with high Ca content (figure 9C), which probably reduces abrasion during feeding. All these features render the structural and chemical complexity of radular teeth. The mechanical property hardness *H* measures a material’s resistance to plastic deformation under indentation or abrasion, while the Young’s modulus *E* quantifies material stiffness, reflecting the relationship between stress and strain. Stiffness specifically influences a tooth’s ability to transfer forces, whereas hardness rather influences the wear resistance.

The values of *E* and *H*, along with gradients within individual teeth and across rows, are closely tied to ecological niches (e.g. feeding on rock, plant surfaces or sand) and tooth functions (e.g. loosening food from surfaces or gathering particles) [36,37]. In general, species feeding on soft ingesta, such as algae on soft substrates (e.g. sand or mud), possess softer, more flexible teeth with *E *≤ 8 GPa and *H *≤ 1 GPa, lacking pronounced gradients from the tooth base to cusp [34]. These teeth, though less capable of withstanding high forces, deform and twist to avoid structural failure when encountering larger obstacles. Their flexibility allows a broader surface area for substrate interaction, facilitating particle collection [33,34]. Species foraging on solid ingesta, such as algae on rocks or prey with hard structures, have significantly stiffer and harder teeth [36,37]. In Polyplacophora, the teeth exhibit *E* values of 30−130 GPa and *H* values of 4−12 GPa [26,29,55]. In Patellogastropoda, the dominant teeth show *E* values of 52−150 GPa and *H* values of 3−7 GPa [50,58]. In Vetigastropoda (*Megathura crenulata*), teeth demonstrate *E* values of approximately 16 GPa [30]. Solid-ingesta feeders often exhibit pronounced gradients in *E* and *H* along individual teeth, with the cusp being the hardest and stiffest, followed by the stylus and the base [26,29–32,34,50,55,58,60,83,84]. These gradients allow cusps to puncture or scrape solid surfaces while minimizing wear or failure, and the softer bases act as shock absorbers. In some taxa, all teeth in a row exhibit similar mechanical properties, signifying a ‘monofunctional radula’ [34,36,37,41,60]. Conversely, others, like certain paludomid gastropods and Polyplacophora, show inter-tooth gradients, with central teeth being stiffer and harder than laterals and marginals, supporting a ‘multifunctional radula’ [29,31,32,34,36,37,83,84].

The mechanical property values measured in *V. turrita* cover a large range (figure 9A); the lateral tooth II shows values in the range of solid substrate feeders, whereas the central tooth and the outer marginal teeth are rather in the range of soft-substrate feeders. The teeth of *V. turrita* could only be tested in dry conditions because of their geometry, even though teeth are naturally used in a wet state. Typically, *H* and *E* values of wet materials are lower than those of their dry counterparts, as dry materials exhibit reduced fracture toughness. For instance, this has been reported for chiton radular teeth, where wet teeth were found to be 15% softer and more flexible [26]. Therefore, we suggest that the *E* and *H* values of teeth in their native wet conditions would probably be lower.

The mechanical property values in *V. turrita* teeth (figure 9A) are increased at the regions that were previously [14] found to interact frequently with the ingesta (the lateral tooth II, the inner marginal teeth, the lateral edge of the lateral tooth I), whereas the regions that did not interact with the substrate were rather soft and more flexible (the central teeth, the outer marginal teeth, the medial side of the lateral tooth I, the thickenings of the membrane I and II). This verifies our hypothesis (a). It renders the rhipidoglossan radula as an example of a multifunctional radula with harder and stiffer teeth, probably used for loosening the food from the substrate (lateral teeth II, the lateral side of the lateral tooth I), and with softer and more flexible teeth, probably brushing food particles (marginal teeth). The softer and more flexible central tooth, together with the medial side of the broad lateral tooth I, as well as the thickenings of the membrane I and II, seems to act as joints, which all together span the radula during forage intake and keep teeth in an upright position.

### 4.3. Origin of mechanical property gradients

In the past, several mechanisms have been found to contribute to increasing the *E* and *H* values of radular teeth.

One mechanism would be mineralization; the radular teeth of Polyplacophora and Patellogastropoda exhibit significant mineral incorporation, with Fe and Si (silica, goethite, iron oxides, apatite) embedded in the chitin matrix (e.g. [11,27,29,36,37,49,50,58,75,85,86]). These highly mineralized teeth are among the stiffest biomaterials, with *E* values ranging from 30 to 130 GPa and *H* values from 4 to 12 GPa in Polyplacophora [29,85]. Dominant teeth in *Patella vulgata* can exceed these values (*E*: 52−150 GPa, *H*: 3−7 GPa) [58,86].

While most molluscs have less mineralized teeth, previous studies found Fe present in small amounts (less than or equal to 1 atomic %) in various snail taxa, alongside Si (less than or equal to 2 atomic %), possibly in the form of silica [29,32,38,60,63,87,88]. Other elements, such as P, appear in tooth cores as iron phosphate or calcium phosphate (apatite), often associated with Mg and F, enhancing *E* and *H* values [85,86,89–91].

In *V. turrita*, we found several elements in the inner tooth structure. Their proportions and distributions were similar to the ones of the previously described elemental composition of *V. turrita* radulae [53,63]. PPt and Si were present in all teeth, but in small proportions. This indicates that apatite and silica mineralization might not be important contributors to the mechanical properties in this species. Fe was detected in small amounts; however, exclusively in the lateral teeth II (figure 9D). This indicates that Fe might be important, either in crystalline form or, probably, as a cross-linker for proteins. Additionally, we found Ca to be present inside the teeth (figure 9B), correlating with *E* and *H*. Whether Ca is present as a mineral or as cross-linker awaits further investigations as the EDX method does not allow testing for this.

In teeth with low internal mineralization, a thin, hard, Si- or Ca-rich coating on the surface was documented for Nudibranchia, Paludomidae, Cephalaspidea and Cephalopoda [15,32,35–38,60,61]. In *V. turrita* (figure 9C), we detected a high content of Ca on the surfaces of the lateral tooth II, the medial side of the lateral tooth I and the cusps of the marginal teeth, verifying our hypothesis (c). This may help reduce abrasion during interactions with the substrate. A higher content of minerals, incorporating elements, such as S, Cl, Ca and Si, in the outer tooth matrix in the form of granules was previously described for neritid teeth of *Nerita atramentosa* [92]. This is comparable to the coatings detected previously, but also to the ferrihydrite layer in chiton teeth [26,45,93,94].

Additionally, ions, such as Ca, Cu, Fe, Mg, Si and Zn, can act as cross-linkers in chitinous materials, increasing mechanical property values due to their ability to cross-link proteins [30,36,37,53,54]. For instance, in the vetigastropod *Megathura crenulata*, Mg and Ca ions elevate *E* to 16 GPa [30]. These ions, alongside Na and P, which are found in radular teeth of various taxa [28,36,37,53,87,95], probably contribute to the protein cross-linking and structural integrity [96]—a similar mechanism to the insect cuticle [97]. In *V. turrita*, we detected Fe, Mg and Si in small proportions and Ca in larger proportions (figure 9D), correlating with *E* and *H*—which verified our hypothesis (b). Whether these elements are present in the form of ions awaits further investigations.

The extent of tanning and protein abundance also influences mechanical properties, paralleling findings in arthropod cuticle. Autofluorescence signals from CLSM used to characterize arthropod material composition [66–69] were recently applied to radulae [15,32,36–38,60,61]. In the radular teeth of *V. turrita*, we found that the autofluorescence signals correlate with the following material properties (figure 9D): (i) stiffer and harder material (the lateral sides of the lateral teeth I, the thickenings of the membrane I and II, the styli and lateral region of the cusps of the lateral teeth II and the styli of the inner marginal teeth) produced a red signal as answer to the lasers of 555 and 639 nm wavelength. These structures are probably sclerotized, similar to some regions of the insect cuticle. (ii) Softer and more flexible material (the central teeth, the medial sides of the lateral teeth I, the bases of the lateral teeth II, the bases of the outer marginal teeth) corresponded to a blue signal (405 nm excitation). Similar to insects, these regions probably contain elastic proteins or unsclerotized chitin. (iii) Regions with intermediate mechanical property values (the middle region of the lateral teeth I, the styli of the outer marginal teeth) responded to the 488 nm wavelength laser (translated to green). Potentially, these regions are weakly sclerotized, similar to the situation in the insect cuticle. (iv) Regions with high Ca content in the coating (the lateral sides of the lateral teeth I, the thickenings of the membrane I and II, the styli and lateral region of the cusps of the lateral teeth II and the styli of the inner marginal teeth) exhibited autofluorescence in response to the 405 nm laser (translated to blue), similar to the situation in crustacean cuticle [67–69] and in other mollusc radulae with high inorganic content [36–38,60,61].

### 4.4. Radular morphology

The precise tooth morphology is known to be influenced by feeding ecology. Variations in tooth size, shape, aspect ratios, uniformity or divergence within a row and tooth distribution on the membrane are often related to the adaptations to food or to substrate (e.g. [4,33,98–106]). Additionally, tooth morphologies reflect different functions as broader teeth are rather involved in loosening food from the substrate, whereas pointy teeth rather pierce or cut tissues (e.g. [15,24,25,33,106–108]). In *V. turrita*, we determined large differences in sizes and morphologies between the teeth (see [14] for a discussion on tooth morphology). In a future study, which will involve three-dimensional models, the mechanical properties and the arrangement of the teeth on the membrane, the influence of the mechanical property gradients on the stress and strain distribution in the teeth will be tested by finite element analysis.

## 5. Conclusion

The findings underscore the radula of *Vittina turrita*, a snail species foraging from rocky surfaces, as a complex composite structure. The radula’s architecture, with chitin fibres interwoven with proteins and arranged perpendicularly to a thin, hard surface coating containing high calcium content, seems to play a critical role in mitigating fracture propagation and reducing wear. This organized fibre orientation is reminiscent of arrangements observed in other molluscs such as limpets and chitons. Moreover, the mechanical property gradients across the teeth—characterized by varying Young’s modulus and hardness—highlight a multifunctional system. Harder, stiffer tooth regions are probably optimized to loosen food from substrates, while softer, more flexible areas either serve as shock absorbers or facilitate particle collection. These gradients result from a synergistic interplay of mechanisms including a small degree of mineralization and ion cross-linking, and differential tanning, each contributing to the overall structural integrity. This study, in connection with past literature, illustrates that the structure and chemistry of the radula reflect the mechanical demands imposed by the specific ecological niches and feeding strategy. Future investigations, particularly those employing three-dimensional modelling and analysis in native wet conditions, will further clarify how these gradients and morphologies interplay to enhance radular function.

## Data Availability

All data can be found in the electronic supplementary material [109].
